# Considering uncertainties expands the lower tail of maize yield projections

**DOI:** 10.1371/journal.pone.0259180

**Published:** 2021-11-18

**Authors:** Haochen Ye, Robert E. Nicholas, Samantha Roth, Klaus Keller

**Affiliations:** 1 Department of Geosciences, The Pennsylvania State University, University Park, Pennsylvania, United States of America; 2 Earth and Environmental Systems Institute, The Pennsylvania State University, University Park, Pennsylvania, United States of America; 3 Department of Meteorology and Atmospheric Science, The Pennsylvania State University, University Park, Pennsylvania, United States of America; 4 Department of Statistics, the Pennsylvania State University, University Park, Pennsylvania, United States of America; 5 Institute for Computational and Data Sciences, the Pennsylvania State University, University Park, Pennsylvania, United States of America; 6 Thayer School of Engineering, Dartmouth College, Hanover, New Hampshire, United States of America; Texas A&M University, UNITED STATES

## Abstract

Crop yields are sensitive to extreme weather events. Improving the understanding of the mechanisms and the drivers of the projection uncertainties can help to improve decisions. Previous studies have provided important insights, but often sample only a small subset of potentially important uncertainties. Here we expand on a previous statistical modeling approach by refining the analyses of two uncertainty sources. Specifically, we assess the effects of uncertainties surrounding crop-yield model parameters and climate forcings on projected crop yield. We focus on maize yield projections in the eastern U.S.in this century. We quantify how considering more uncertainties expands the lower tail of yield projections. We characterized the relative importance of each uncertainty source and show that the uncertainty surrounding yield model parameters is the main driver of yield projection uncertainty.

## 1. Introduction

Increasing greenhouse gas concentrations lead to warmer climates and more frequent extreme weather events [1]. Climate change poses threats to many economic sectors. For example, agricultural yields can be highly sensitive to temperature and precipitation change, raising concerns about food security [2].

The net impact of climate change on agriculture is highly uncertain due to our limited knowledge about drivers of yield anomalies and future climates [3–5]. Improving our understanding of climate change impacts on crop yields and quantifying the surrounding uncertainties is a potentially important avenue to improve decisions.

Statistical models are often used to represent the weather-crop yield relationship and to produce yield projections [6]. Statistical models empirically relate historical weather data and crop yield observations. Another approach to study the weather-crop yield relationship uses process-based dynamical simulations, which simulate the physiological processes of crop growth. Compared with dynamical models, statistical models have lower computation cost. This drastically simplifies the uncertainty assessments surrounding yield projections [6, 7].

Schlenker and Roberts (2009) uses statistical models to identify a nonlinear effect of temperature on crop yields and shows that crop yields increase modestly as temperature increases and decrease sharply once temperature exceeds a particular threshold [4]. Based on this nonlinear weather-yield relationship, Schlenker and Roberts (2009) projects a maize yield decrease of 63%-82% with 95% confidence level by the end of this century (2070–2099) under the most extreme warming scenario considered [4].

Previous studies provide valuable insights about weather impacts on crop yields, but they often sample a rather small subset of potentially important uncertainties. Some studies apply a simple “delta method” to climate projections [4, 8]. In other words, these studies approximate the future climate distribution by a linear shift of the past climate. This assumption is inconsistent with observations that suggest that the shape of summer temperature distributions has already changed in the past [9]. More recent studies adopt more complex and realistic climate forcings when projecting crop yields [10, 11]. Burke et al (2015) reports the 95% confidence intervals of maize yields projections in multiple climate forcings based on a relatively simple statistical model [10]. Keane and Neal (2018) lists the range of yield projections under 19 general circulation models (GCM) and three representative concentration pathway (RCP) emission scenarios [11]. These studies are, however, mostly silent on the relative importance of different uncertainty sources.

Here we expand on the statistical analysis of Schlenker and Roberts (2009) by incorporating and quantifying the effects as well as the relative importance of two main uncertainty sources on yield projections [4]: (i) uncertainties surrounding model parameters and (ii) climate forcings. We focus on maize as it is widely grown in most U.S. states and has high data availability. Following past studies [4, 8, 12], we consider six weather variables in a simple regression model and allow each variable to be either neglected or included in a linear or quadratic term in the model. To approximate the effects of model parameter uncertainty, we sample model parameters that pass a simple pre-calibration test [13, 14] based on observation data and the best estimates of yield hindcasts. To sample the climate forcing uncertainty, we use an ensemble of downscaled climate products to represent sampled climate conditions in future. We project the yield distribution based on sampled model parameters and climate forcings. Finally, we quantify the relative importance of these uncertainty sources by using a cumulative uncertainty approach based on the standard deviations when considering different uncertainty sources [15].

We address two main questions: (i) How does the incorporation of different uncertainties change the maize yield projection? (ii) What is the relative importance of each uncertainty source? The remaining text introduces the chosen yield data as well as climate data (section 2), describes the process of model regression and uncertainty analysis in detail (section 3), reports the main results (section 4), discusses methods and results (section 6) as well as caveats and limitations (section 7). The last section summarizes the conclusions and points to research needs.

## 2. Data

We collect county-level annual maize yield data from the United States Department of Agriculture [16]. We focus on 24 states in the eastern U.S. because they often rely more on precipitation than irrigation [4]. These yield data are reported as unit yield per growing area (bushel/acre) along with growing area in each county. We drop the counties with unreported data. We calculate the annual average yields for the entire study region weighted by reported growing areas.

We use METDATA historical climate data, a relatively high-spatial resolution (4km*4km) daily surface meteorological data product covering the contiguous U.S. [17]. We choose the historical study period from 1979 to 2018. We consider five weather variables based on previous research: maximum temperature, minimum temperature, precipitation, maximum relative humidity and minimum relative humidity [12]. We use weather data within the maize growing season each year defined as a 6-month interval after the 21-day moving average temperature reaches 10°C [12].

For the climate projections, we use MACAv2-METDATA [18]. This dataset uses the Multivariate Adaptive Constructed Analogs (MACA) statistical method to downscale GCMs bias corrected by METDATA observations. We analyze the 2019–2099 period from these projections to extend the observed data. We focus on two 30-year time windows to represent the near (2020–2049) and far future (2070–2099). Similar to how we dealt with the METDATA observations, we extract the same daily weather variables within the maize growing season. We choose to use projections following the business-as-usual RCP8.5 scenario [19] for comparability with other studies [4, 11, 12].

We aggregate all weather data to the county level based on each grid center’s longitude and latitude [8]. For each county, we find the grids whose centers fall inside the county boundary. We take the mean of these grids to serve as county level average weather data.

To capture some measure of the uncertainty in climate forcing, we use an ensemble of MACAv2-METDATA projections that comprises 18 climate projections based on different Coupled Model Intercomparison Project v5 (CMIP5) models [20]. These climate projections differ considerably. We consider these ensemble members as equally likely, as there is little evidence that one model outperforms the others in terms of root-mean-square errors (RMSE) over space and time compared with observations [10].

To simplify the comparison with previous studies, we also apply the delta downscaling method for climate projection as a scenario without considering climate forcing uncertainty [21]. We realize that this is a strong approximation and use this as an idealized scenario for comparison only [8]. For each 30-year time window, we calculate the mean difference or ratio of each weather variable between MACAv2-METDATA projection and hindcast, and then shift the METDATA observations of 1981–2010 to generate a new climate. Specifically, for each 30-year projection period, we calculate the 30-year mean value for each weather variable in each MACAv2-METDATA projection dataset. We then use the multi-model ensemble mean from the 18 climate projections as the mean projection for each variable [22]. We further calculate each variable’s 30-year hindcast mean value from MACAv2-METDATA hindcast dataset for a time window of 1981–2010. We shift the observational temperatures linearly based on the absolute difference between projection mean and hindcast mean, and multiply the observational precipitation and relative humidity proportionally based on the ratio between projection mean and hindcast mean.

## 3. Methods

**Fig 1:**
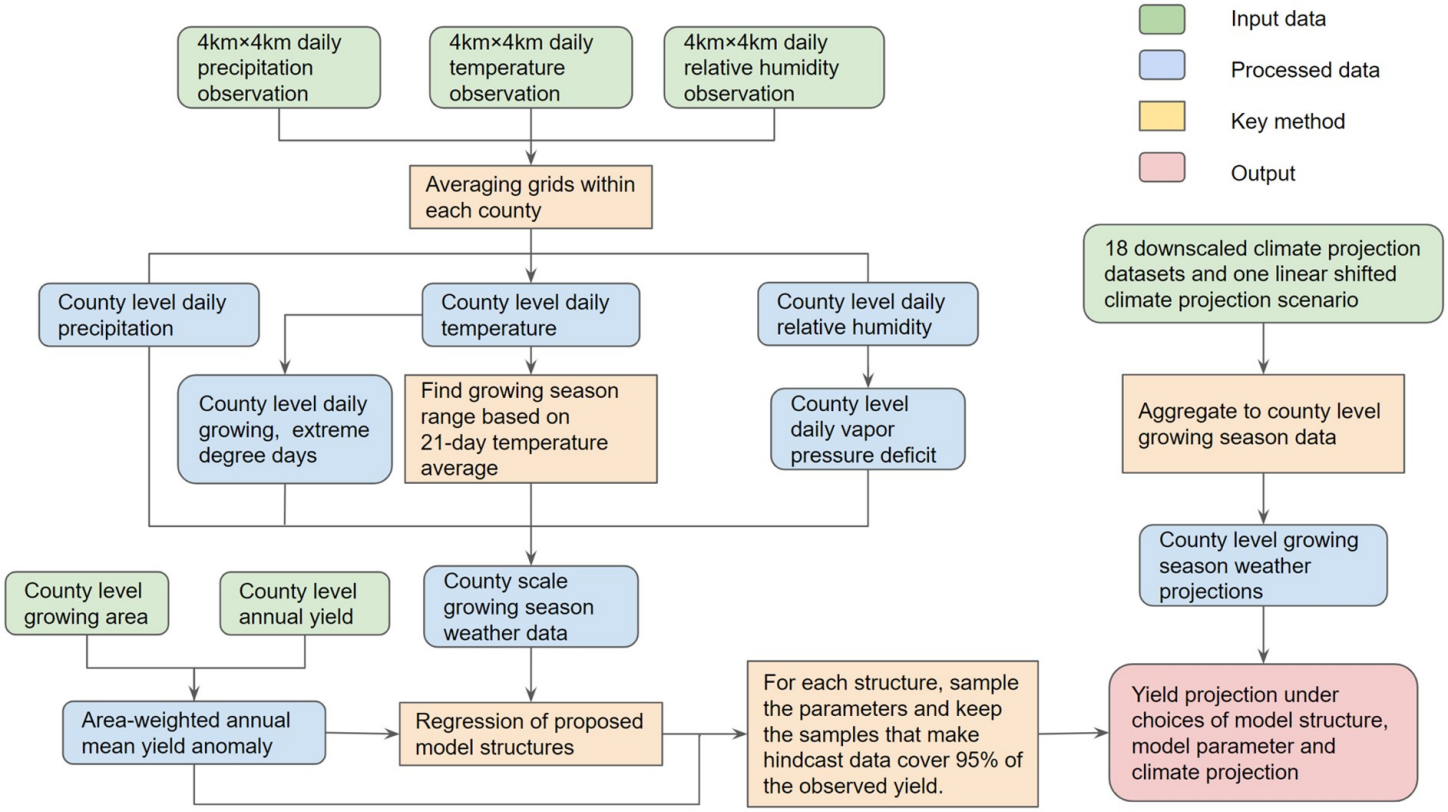
Analysis flow diagram. We adopt county level weather data and yield data to model the weather impact on maize yields. We consider parameter uncertainty through a pre-calibration method and climate forcing uncertainty through an ensemble of downscaled climate products.

The design of the analysis is illustrated in the flow diagram (Fig 1). We consider six weather variables that previous work identified as important based on the five weather variables reported in the historical climate data: maximum temperature, minimum temperature, precipitation, vapor pressure deficit (VPD) calculated by temperature and relative humidity, growing degree days (GDD) and extreme degree days (EDD) calculated by temperature [12].

We calculate VPD (in hPa) using Eq (1), where T_mean_ is the average of maximum and minimum temperature in degrees Celsius, and RH_mean_ is the average of maximum and minimum humidity:

$$VPD=6.112\times e^{\frac{17.269\;\times \;\text{Tmean}}{\text{Tmean}-237.3}}\times \left(1-RHmean\right).$$ (1)

We adopt 10°C to 29°C as maize’s growing temperature range and use it to calculate GDD and EDD [4]. A simple estimation method of GDD and EDD follows Eqs (2) and (3) based on daily maximum and minimum temperature [23]:

$$GDD=\frac{Tmax\;+\;Tmin}{2}-10,$$ (2)

and

$$EDD=\frac{Tmax\;+\;Tmin}{2}-29.$$ (3)

To calculate GDD, we treat any temperature higher than 29°C as 29°C, and any temperature lower than 10°C as 10°C. Similarly, to calculate EDD, we treat any temperature lower than 29°C as 29°C.

We analyze a set of model structures and determine the best choice of model structure by minimizing cross validation errors. Specifically, for each variable, we allow the model to include an up-to-quadratic relationship. This means the model may include both quadratic and linear terms, only the linear term, or nothing. The full model is shown in Eq (4).


$$yield=\beta_{1}GDD+\beta_{2}GDD^{2}+\beta_{3}EDD+\beta_{4}EDD^{2}+\beta_{5}Tmax+\beta_{6}Tmax^{2}+\beta_{7}Tmin+\beta_{8}Tmin^{2}+\beta_{9}Pr+\beta_{1}{}_{0}Pr^{2}+\beta_{1}{}_{1}VPD+\beta_{1}{}_{2}VPD^{2}+\beta_{13}.$$ (4)

For each model structure, we apply ten-fold cross validation. We divide the observation data into ten equally sized groups, and we train the model using data from nine groups and test the hindcast performance of the last group. We calculate the RMSE for the test data to assess each model’s predictive skill. We repeat this process for each group and calculate the cross-validation error as the mean of ten RMSE calculations. We adopt the model with the smallest cross-validation error as the best model to estimate the yield hindcasts (Eq 5). We treat this model (Eq 5) as a reference model to represent the common approach and to calculate the yield anomalies.


$$yield=\beta_{1}GDD+\beta_{2}GDD^{2}+\beta_{3}EDD+\beta_{4}Tmin+\beta_{5}Tmin^{2}+\beta_{6}Pr+\beta_{7}Pr^{2}+\beta_{8}VPD+\beta_{9}VPD^{2}+\beta_{1}{}_{0}.$$ (5)

We transform the yield data into anomalies based on the reference model (Eq 5). In order to reduce the influence of other factors than weather, we include additional fixed effect terms in the reference model (Eq 5) and estimate these fixed effects. The temporal fixed effects capture factors that are approximately constant in space such as technology trend, market price and CO_2_ concentration. The spatial fixed effects approximate the effects of factors that are approximately constant in time such as local soil quality. Following previous work, we subtract the best estimates of temporal fixed effects in each year from yield observations [4]. We do not subtract the spatial effects because we are not specifically focusing on a particular region and we work in anomalies space. We then normalize the yield anomalies by subtracting the area-weighted mean yield so that the historical mean yield anomaly is zero. In the analyses of parameter uncertainties and yield projections, the calculations are based on normalized yield anomalies.

We use a simple pre-calibration approach to sample the model parameter uncertainty [13, 14]. The goal of pre-calibration is to characterize the parameter uncertainties and to drop unrealistic parameter samples by comparing the hindcasts with observations. The pre-calibration approach can provide several advantages. For example, it does not require a specific functional form for the parameter estimates. In addition, it provides a simple and straightforward way to sample the parameters with consideration of parameter interactions. Instead of directly applying the best model, we consider the full model shown in Eq (4) for the pre-calibration process. The best model treats the parameters that are excluded from the full model as zero. Hence the uncertainties of these parameters are neglected if the uncertainty characterization is based on the best model.

For each parameter, we specify a wide uniform range around the best estimate to sample from. The range width for all parameters (except the EDD terms) is twenty standard deviations around the best estimate of the full model. For EDD terms we use 50 standard deviations in order to cover the best estimates of the best model. We use Latin hypercube sampling to draw 10^10^ samples within this range without considering correlation [24]. We define a plausible hindcast range to be a symmetric band around the best estimate of hindcasts from Eq (4). We adopt the width of this band as the minimum width that enables the band to cover 95% of the area-weighted annual yield anomaly observation. We accept a parameter sample if the yield hindcast falls within this plausible range. We can observe the correlation between each pair of parameters from a two-dimensional heat map of accepted parameter samples after the pre-calibration (S1 and S2 Figs). Altogether we accept 19,231 samples. We do not find strong evidence that the yield projections change drastically when using more samples (S3 Fig). We hence consider the sample size of 10^10^ a reasonable approximation. This pre-calibration approach can certainly be refined [25], but it provides a simple and intuitive benchmark.

To assess the yield projection uncertainty, we sample climate forcing and model parameter uncertainties. Specifically, we sample 19 climate forcings and 19,231 accepted parameter samples. As a reference, we use the linear shifted climate projection from the delta method and the best estimates of parameters to represent the scenario without considering either uncertainty. For each 30-year interval, we calculate the average yield anomaly distribution while sampling different uncertainties.

To quantify the importance of the two uncertainty sources, we employ a cumulative uncertainty approach [15]. We use the standard deviation of the yield anomaly distribution from each of the 30-year windows to represent the uncertainties. The cumulative uncertainty approach decomposes the total uncertainty into individual uncertainty sources by calculating the uncertainty at different stages. Again, this method can certainly be refined [26], but it can provide some useful initial insights [15]. A stage is defined as a choice of considering certain uncertainty sources. For each stage, we first calculate the conditional cumulative uncertainty by fixing the factor(s) after this stage and varying the factor(s) up to this stage. For example, our first stage begins with varying model parameters while fixing the climate forcing. Under each choice of climate forcing, we calculate the standard deviation from the yield anomaly distribution considering parameter uncertainty. Then the marginal cumulative uncertainty of this stage is the mean of conditional cumulative uncertainty when choosing different factors after this stage. In our case, it is the mean of the standard deviations when choosing different climate forcings. The marginal cumulative uncertainty represents the cumulative uncertainty up to this stage. In this study, we list all three stages with their marginal cumulative uncertainty. The three stages are: (i) a stage considering only parameter uncertainty (ii) a stage considering only climate projection uncertainty and (iii) a stage considering both uncertainties.

## 4. Results

**Fig 2:**
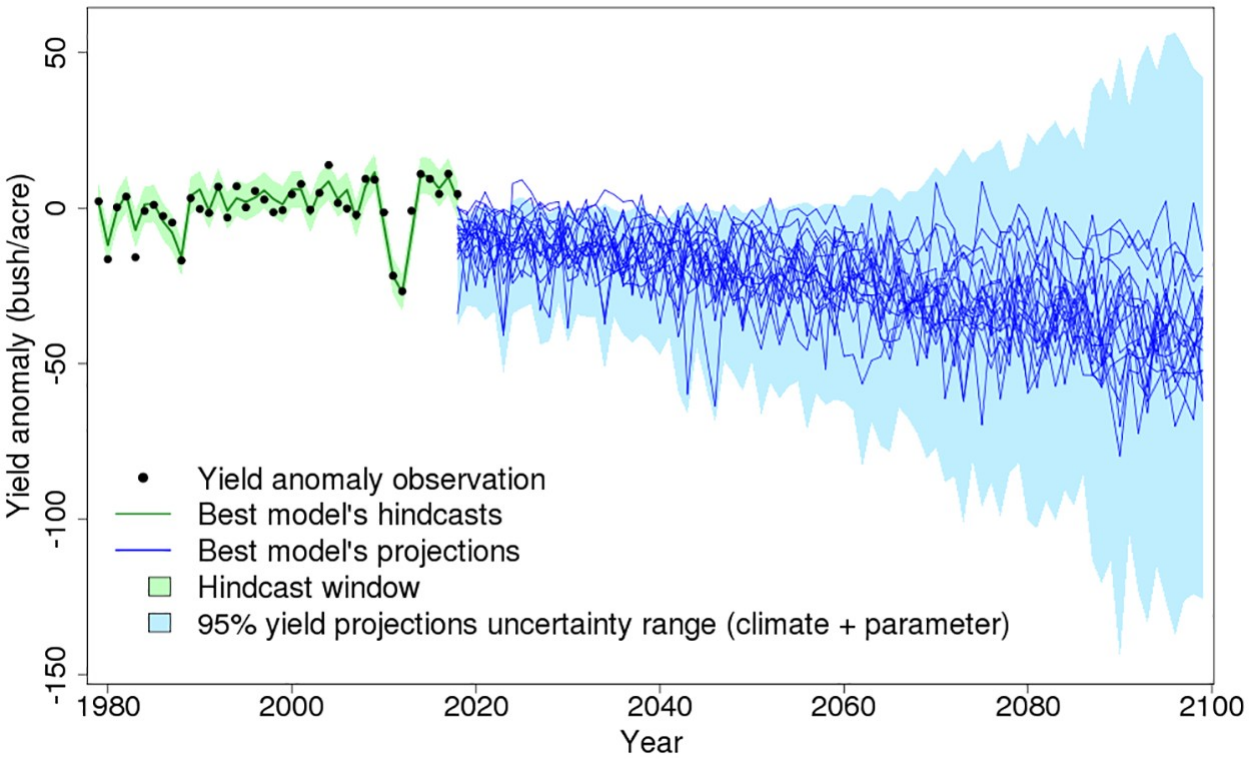
Annual mean yield hindcasts and projections under different methodological choices. The black dots represent the area-weighted average annual yield observations. The green line is the best estimate of yield hindcast based on the model with the least cross-validation error. The deep blue lines are best estimates of this model for 18 different climate projections. Adding the effects of considered parameter uncertainty allows a pre-calibration to cover 95% of the observed yield data (light green area). The total effects of the considered uncertainties (climate forcings and parameters) expand the projection with a much wider 95% uncertainty range (light blue area).

We estimate the best yield hindcasts and projections based on the selected model with the least cross-validation error shown in Eq (5) (the green and blue lines in Fig 2). Compared with the full model in Eq (4), the best model does not include maximum temperature (T_max_) terms and the quadratic extreme degree days (EDD) term.

Similar to previous studies, increasing growing degree days (GDD) increases maize yield while increasing extreme degree days (EDD) decreases maize yield. While the best model and the full model have similar hindcast skills, some of the parameter estimates are different, especially linear EDD term and linear T_max_ term (Table 1). This suggests that these two models may have different yield projections under extreme high temperatures.

**Table 1:** List of model estimates and parameter sampling range.

Terms (units)	Best model best estimate	Full model best estimate	Full model standard error	Sampling range
**Intercept**	-193	-222	21.0	-432, -12.0
**GDD (degree day)**	0.315	0.355	1.60e-2	0.195, 0.515
**GDD**^**2**^ **(square degree day)**	-1.16e-4	-1.18e-4	5.01e-6	-6.79e-4, -1.68e-4
**EDD (degree day)**	-0.172	-0.272	6.80e-3	-0.612, 6.80e-2
**EDD**^**2**^ **(square degree day)**	0	3.48e-4	2.04e-5	-6.72e-4, 1.37e-3
**Tmax (°C)**	0	0.809	2.38	-23.0, 24.6
**Tmax**^**2**^ **(square °C)**	0	-6.05e-2	5.04e-2	-0.565, 0.444
**Tmin (°C)**	-21.7	-21.8	1.39	-35.7, -7.9
**Tmin**^**2**^ **(square °C)**	1.42	1.35	5.92e-2	0.76, 1.94
**Pr (mm)**	9.36e-2	8.73e-2	4.91e-3	3.82e-2, 0.136
**Pr**^**2**^ **(square mm)**	-8.22e-5	-7.92e-5	3.52e-6	-1.14e-4, -4.4e-5
**VPD (hPa)**	0.681	0.693	6.35e-2	5.80e-2, 1.33
**VPD**^**2**^ **(square hPa)**	-2.75e-3	-2.75e-3	3.00e-4	-5.75e-3, 2.5e-4

**Fig 3:**
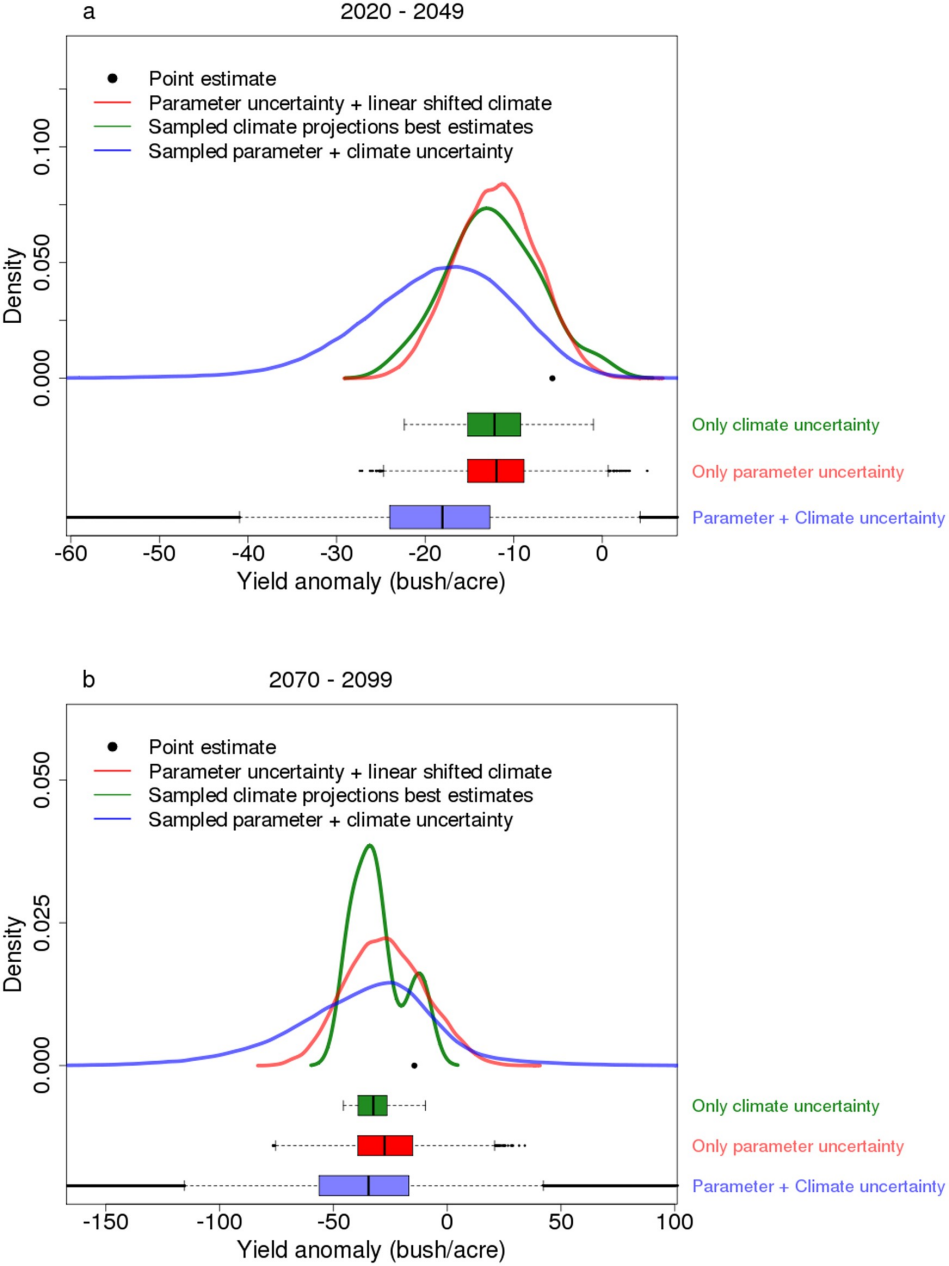
Marginal distributions of 30-year mean yield projections for different considerations of uncertainties. **a: 2020–2049 b: 2070–2099** The point labels the point estimate without considering any uncertainty (the best estimate in a linear shifted climate projection); the three solid lines (red, green and blue) are the distributions when considering only parameter uncertainty, only climate forcing uncertainty and both uncertainty sources respectively. The distribution medians are labeled as vertical black lines on the box-whisker plots.

Yield projections change considerably when moving from climate projections derived from the delta method to the more refined downscaling method (Fig 3). One reason for this is that the delta method underestimates the extreme high temperatures (S4 Fig). In this example, the delta method underestimates the projected temperature mean by about 0.7°C. This effect is amplified for EDD, because EDD represents the net effect of the extreme temperatures, which is the tail area of mean temperature distribution (S5 Fig). The increasing extreme high EDD can lead to potentially sharp decreases in yield projections.

The distributions of the accepted pre-calibration parameter samples are much wider than the distributions based on the linear regression results from the best model (S6 Fig). Recall that our approach accepts parameter samples as long as the hindcasts pass the defined plausible band. Many best parameter estimates are not located at the highest density of the accepted pre-calibration samples (S1 and S2 Figs, Table 1).

As expected, adding climate forcing uncertainty to model parameter uncertainty widens the yield projection uncertainty range (Fig 2). The upper bound of the uncertainty range does not considerably increase until around 2060, but the lower bound decreases from about -40 bushel/acre in the near future down to about -150 bushel/acre in the far future. One hypothesis to potentially explain the observed patterns of an increasing upper bound of the uncertainty range in the far future is that some sampled structures are more sensitive to the positive effects of climate change. In this case, the yield projections will be high under a warm but not extreme climate forcing which has high GDD and low EDD. The results in Fig 2 are consistent with this hypothesis: although many model samples have similar hindcast skill passing the green plausible band, their predictive skill will diverge greatly, especially under an unexperienced more extreme future climate.

The best estimates of yield projections from different climate projections miss important information about the possible low yield extremes (blue lines in Fig 2). In the near future, some lines project positive yield anomalies that exceed the upper limit of the 95% uncertainty range. In the far future, though the yield projection uncertainty will have a high upper bound, the best estimates from each climate projection are all negative values. However, in both the near future and far future, these best estimates are much higher than the lower bound of the 95% uncertainty range. This suggests that considering both uncertainty sources will lead to more extremely low yield projections.

Considering model parameter uncertainty widens the probability density function of the yield projections (red lines in Fig 3). Similarly, considering climate forcing uncertainties produces roughly similar distributions (green lines in Fig 3). Considering both uncertainty sources extends the lower tails even further and results in yield distributions with negative skewness (blue lines in Fig 3).

**Fig 4:**
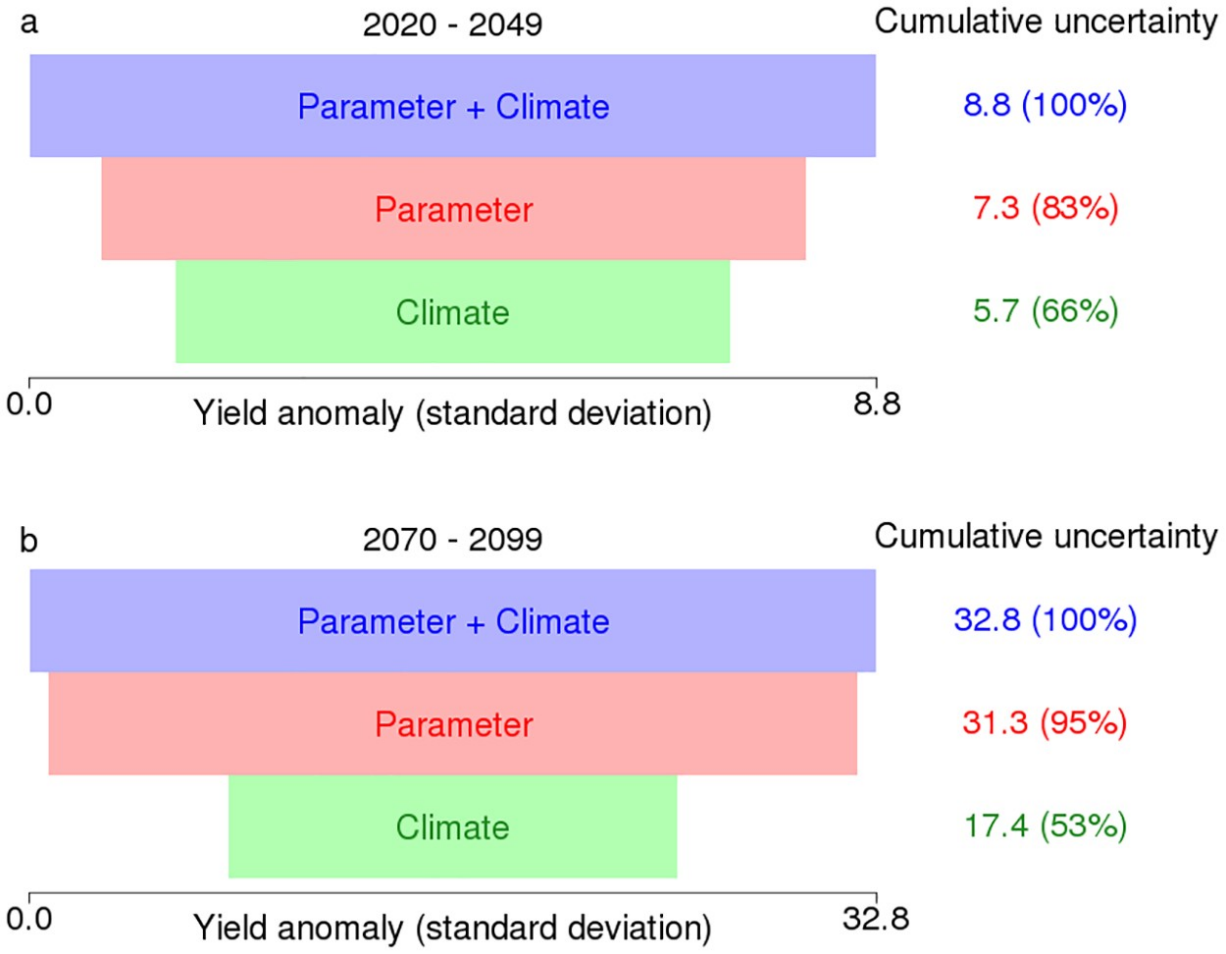
Decomposition of the uncertainty in 30-year mean yield distributions. The uncertainties are measured in yield anomaly standard deviations. Two panels are for two periods. **a**: 2020–2049 **b**: 2070–2099. The percentages are the proportion of the uncertainty cumulatived up to each stage.

Model parameter uncertainty explains more of the variance in yield projections than climate forcing uncertainty (Fig 4). The importance of the parameter uncertainty increases in the far future. In the near future, the stage with only climate forcing uncertainty explains around 66% of total variance, while the stage with only parameter uncertainty explains around 83% of total variance. In the far future, the stage with only climate forcing uncertainty explains around 53% of total variance while the stage with only parameter uncertainty explains around 95% of total variance.

## 5. Discussion

We expand on a well-studied approach to project changes in maize yields to analyze the effects and the relative importance of model parameters and climate uncertainties [4]. The pre-calibration approach provides a conceptually easy approach to analyze the effects of parameter uncertainty. However, it is computationally very demanding. In our case with 13 parameters, the acceptance rate is about one in a million using the current sampling range. The heat map suggests that the prior range is still not wide enough because samples can still be accepted near the boundaries (S1 and S2 Figs). Another potential concern is that some accepted samples project rather extreme yield anomalies in the far future. It is possible for a model to pass the pre-calibration test but project extreme yields under an extreme climate beyond historical climate (S7 Fig). This points to potential problems with the statistical model approach.

We use a cumulative uncertainty decomposition method to quantify the relative importance of each uncertainty source. However, the decomposition result depends on the measure of uncertainty and the order of uncertainty sources to add. The common approach starts with one source and adds another at each stage. Then the contribution of a particular source is the difference of cumulative uncertainties between two successive stages with and without this source. In the results for 2020–2049, if we choose to start with climate forcing uncertainty and then add parameter uncertainty, we would conclude that climate forcing uncertainty explains 66% of total uncertainty and the addition of parameter uncertainty explains 100%-66% = 34% of total uncertainty. If we start with parameter uncertainty and then add climate forcing uncertainty, we would conclude that parameter uncertainty explains 83% of total uncertainty while the addition of climate forcing uncertainty explains 100%-83% = 17% of total uncertainty (Fig 4). The results will also be different if we use another measure of uncertainty such as the range of the distribution. We simply list the uncertainty explained by each stage. More refined variance-based uncertainty decomposition methods are available to quantify the relative importance of each uncertainty source [27]. Sobol’s method considers all the uncertainty sources simultaneously and calculates the variance explained by each individual source as well as each interaction between multiple sources.

## 6. Caveats and limitations

We chose our analysis framework for its conceptual simplicity. The simplicity comes, of course, requires several simplifying assumptions that lead to caveats. Here we mention four examples of these caveats. First, we only adopt a simple statistical model with linear and quadratic terms and neglect key aspects of structural model uncertainty. Second, we only consider a high forcing scenario for climate projection (RCP8.5). Including more forcing scenarios will likely expand the uncertainty in climate forcing. Third, we simply treat the 24 states as a whole and produce mean projections for the entire region. We further assume that the statistical relationship and growing area in each county will hold the same in future. In reality, the adaptation of crops and technology development might change the weather-yield relationship greatly [11]. Last but not least, we adopt a very simple statistical approach. For example, we use a very simple acceptance criterion that does not consider the spatial correlation of yield residuals (S8 Fig). The yield anomalies usually have strong spatio-temporal patterns thus models that can capture and simulate these patterns might be considered more realistic.

## 7. Conclusion

Crop yields are sensitive to climate change. Many studies use statistical models to simulate the weather-yield relationship and estimate the yield projection under climate change. However, previous studies have often been silent on the effects and relative importance of the deep uncertainty surrounding model parameters and climate forcings. We identify important uncertainties in model parameters and climate forcings surrounding yield projections. We incorporate these two uncertainty sources using a statistical approach and apply a simple evaluation method to rank their relative importance. We find that considering these uncertainty sources leads to a yield projection with a wider range, larger variance, and a longer tail of low yield outcomes. By comparing the marginal cumulative uncertainty when considering different uncertainty sources, we conclude that model parameter uncertainty explains more uncertainty than sampled climate forcing uncertainty. Our study can help to inform climate impact assessments and the design of strategies to improve these assessments.

## Supporting information

S1 Fig Heat maps of accepted linear parameter samples based on the full model from the pre-calibration analysis. The black dot is the best estimate based on the full model (Eq 4). The colors illustrate the probability density of the parameters with red area denoting higher and blue area denoting lower probability densities.
(TIF)

S2 Fig Heat map of accepted GDD and EDD parameter samples. This figure is a zoomed-in panel of S1 Fig. The black dot is the best estimate based on the full model. The best estimate does not necessarily locate at the highest density region of the accepted pre-calibration samples.
(TIF)

S3 Fig Convergence of the pre-calibration sampling approach. Shown are the far future yield projection (the blue PDF in Fig 3b) mean and standard deviation change as a function of accepted pre-calibration sample sizes. The solid line represents the mean, and the dashed line represents the standard deviation. Both lines stabilize after around 5,000 samples.
(TIF)

S4 Fig Comparison of temperature distribution in far future under linear shifted climate and downscaled climate projection. Here we pick the MIROC5 model projection from MACAv2-METDATA (the red histograms) [18, 20]. The linear shifted climate underestimates the high temperatures and overestimates the low temperatures. On the box-whisker plots, the vertical black lines are the histogram temperature medians (50% percentile), two ends of the box are 25% percentile and 75% percentile temperatures, and the black points are the outliers outside 1.5 times of the interquartile range (the width of the box).
(TIF)

S5 Fig Comparison of extreme degree day (EDD) distribution in far future under linear shifted climate and downscaled climate projection. The box-whisker plots are the same as Fig 1 except that they are for EDD instead of temperature.
(TIF)

S6 Fig Marginal distributions of each parameter. The black lines are the parameter distributions based on the linear regression result for the best model with the least cross-validation errors. This model does not include the quadratic EDD term and Tmax terms so instead there is a black dashed line at zero in these panels. The red lines are the parameter distributions from the accepted pre-calibration samples. The range of x-axis in each panel is the wide prior range of each parameter.
(TIF)

S7 Fig The full yield anomaly projections uncertainty range. This plot is the same as Fig 2 but with the full yield projections uncertainty range instead of 95% uncertainty range.
(TIF)

S8 Fig County level yield residual of the model hindcast. The yield residuals have strong spatial patterns varying each year. We plot the residual map in 1983 with the most observation data. In future studies, we plan to use spatial models to better account for these spatial patterns.
(TIF)

S9 Fig The predictive skill of a model using only 32 years data. We add eight more years observational data in an update (1979, 1980, 2013–2018). We use these data to test the predictive skill of the old model using 32 years data. The estimated hindcasts given by the old model (blue circles) are close to the hindcasts of the updated model (red line) and the observations (black dots).
(TIF)
