# Supplementary figures and images for: Considering uncertainties expands the lower tail of maize yield projections
Source: PLoS One. 2021 Nov 18;16(11):e0259180. doi: 10.1371/journal.pone.0259180 (PMC8601471; doi:10.1371/journal.pone.0259180)

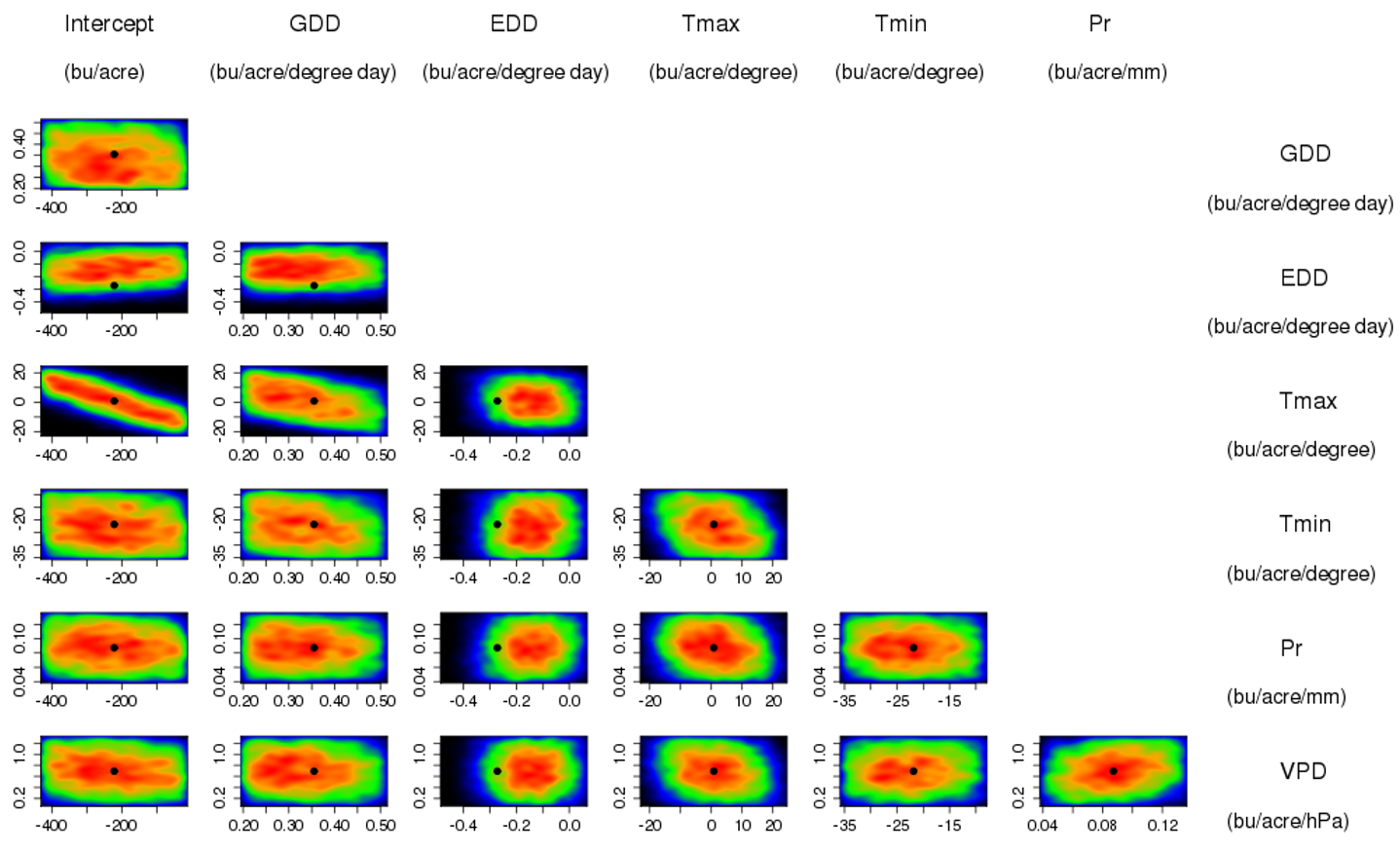

Supplement: S1 Fig — The black dot is the best estimate based on the full model (Eq 4). The colors illustrate the probability density of the parameters with red area denoting higher and blue area denoting lower probability densities. (TIF) [file pone.0259180.s001.tif]

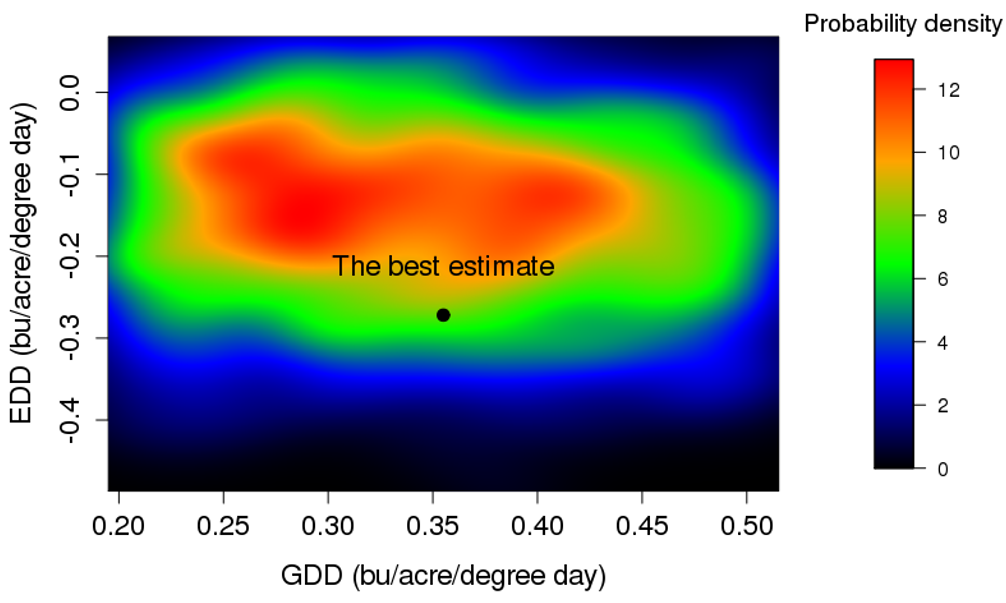

Supplement: S2 Fig — This figure is a zoomed-in panel of S1 Fig. The black dot is the best estimate based on the full model. The best estimate does not necessarily locate at the highest density region of the accepted pre-calibration samples. (TIF) [file pone.0259180.s002.tif]

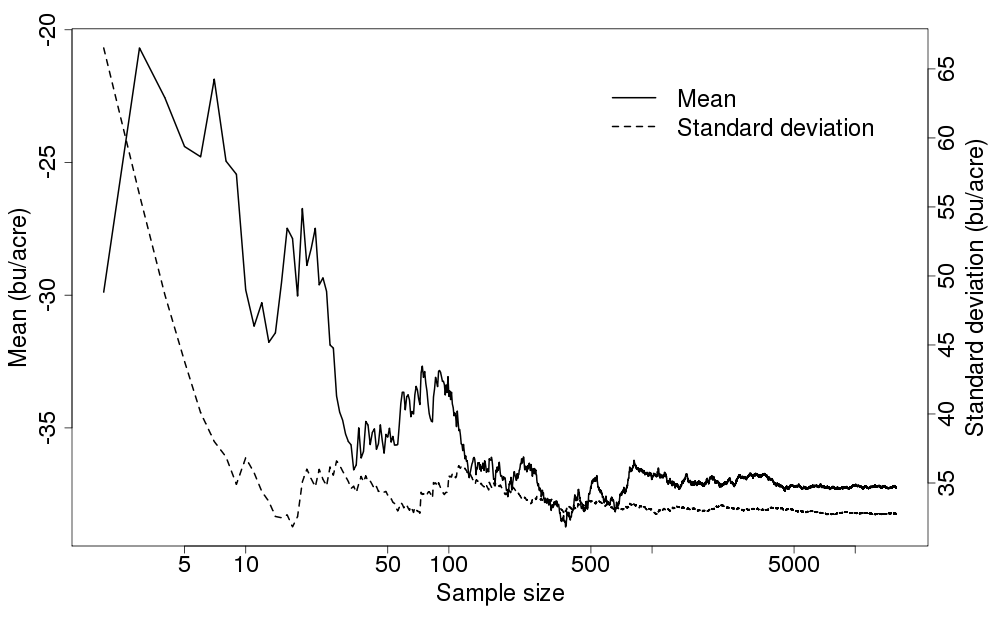

Supplement: S3 Fig — Shown are the far future yield projection (the blue PDF in Fig 3b) mean and standard deviation change as a function of accepted pre-calibration sample sizes. The solid line represents the mean, and the dashed line represents the standard deviation. Both lines stabilize after around 5,000 samples. (TIF) [file pone.0259180.s003.tif]

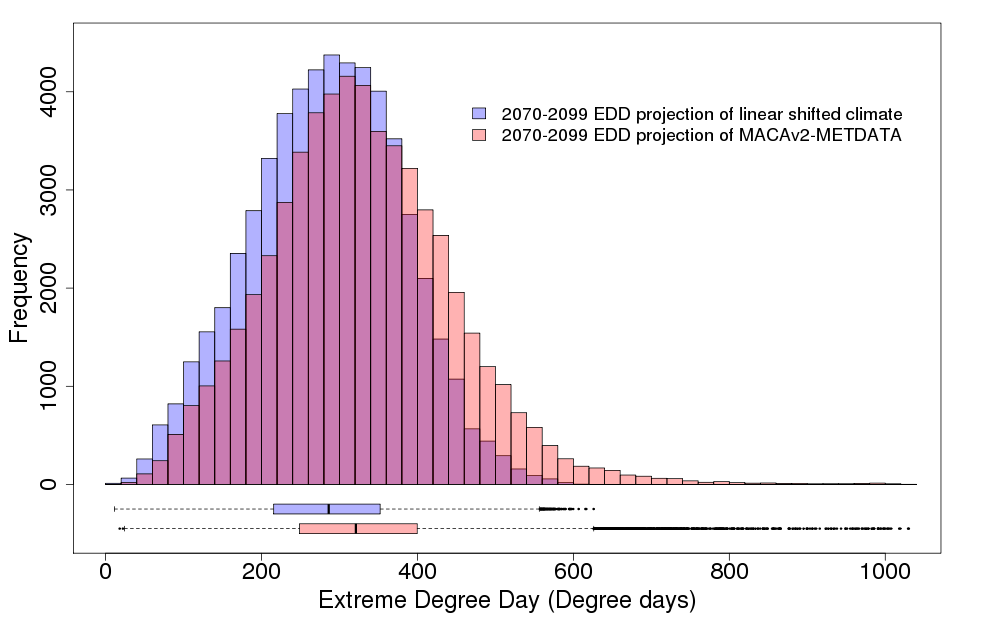

Supplement: S4 Fig — Here we pick the MIROC5 model projection from MACAv2-METDATA (the red histograms) [18, 20]. The linear shifted climate underestimates the high temperatures and overestimates the low temperatures. On the box-whisker plots, the vertical black lines are the histogram temperature medians (50% percentile), two ends of the box are 25% percentile and 75% percentile temperatures, and the black points are the outliers outside 1.5 times of the interquartile range (the width of the box). (TIF) [file pone.0259180.s004.tif]

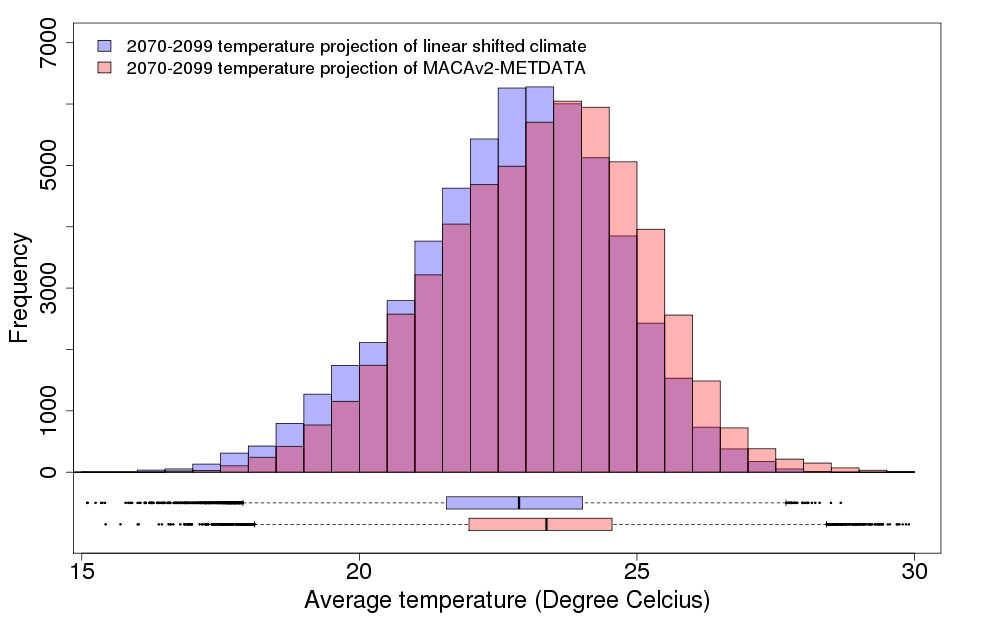

Supplement: S5 Fig — The box-whisker plots are the same as Fig 1 except that they are for EDD instead of temperature. (TIF) [file pone.0259180.s005.tif]

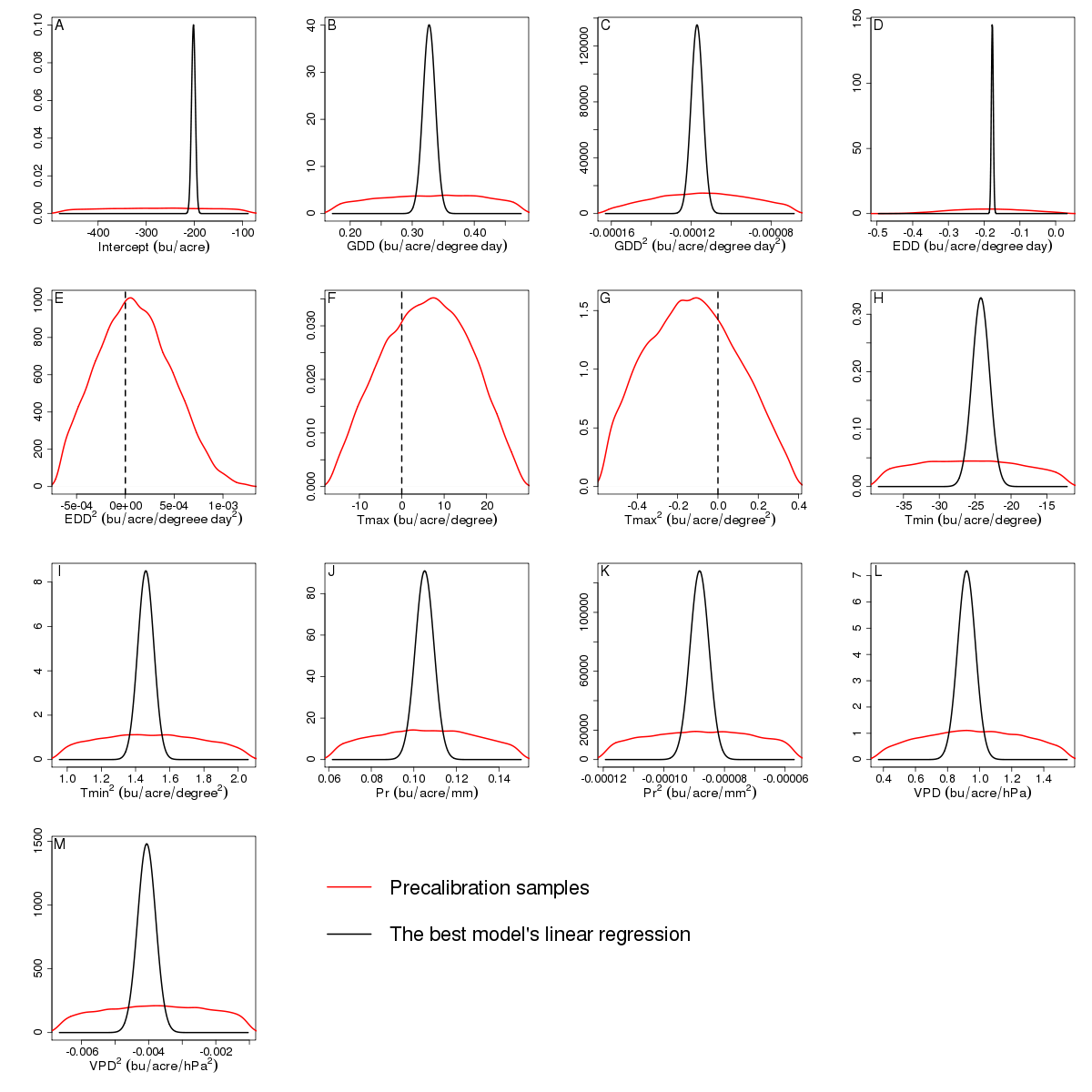

Supplement: S6 Fig — The black lines are the parameter distributions based on the linear regression result for the best model with the least cross-validation errors. This model does not include the quadratic EDD term and Tmax terms so instead there is a black dashed line at zero in these panels. The red lines are the parameter distributions from the accepted pre-calibration samples. The range of x-axis in each panel is the wide prior range of each parameter. (TIF) [file pone.0259180.s006.tif]

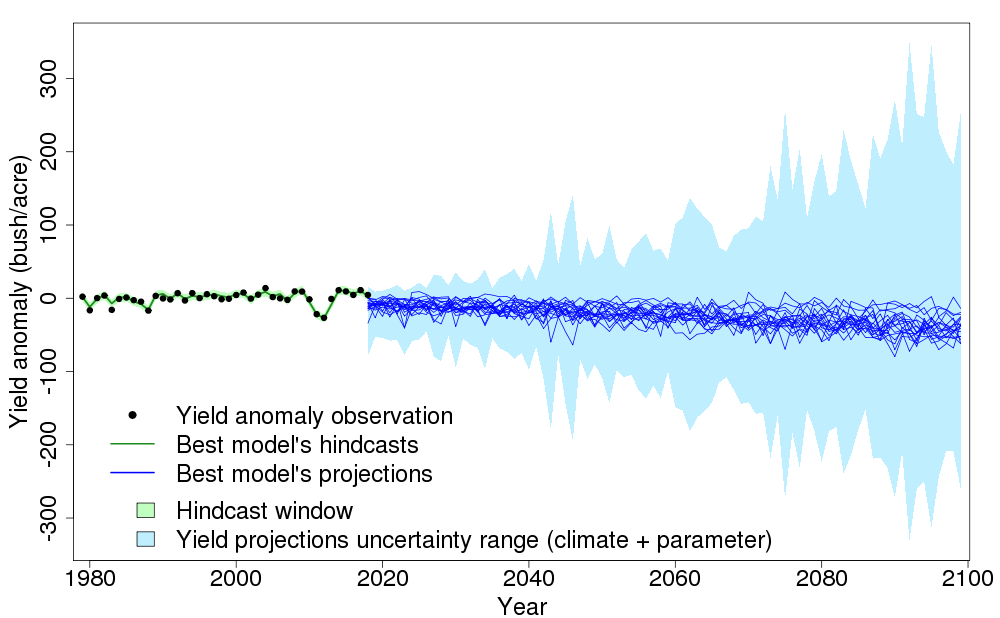

Supplement: S7 Fig — This plot is the same as Fig 2 but with the full yield projections uncertainty range instead of 95% uncertainty range. (TIF) [file pone.0259180.s007.tif]

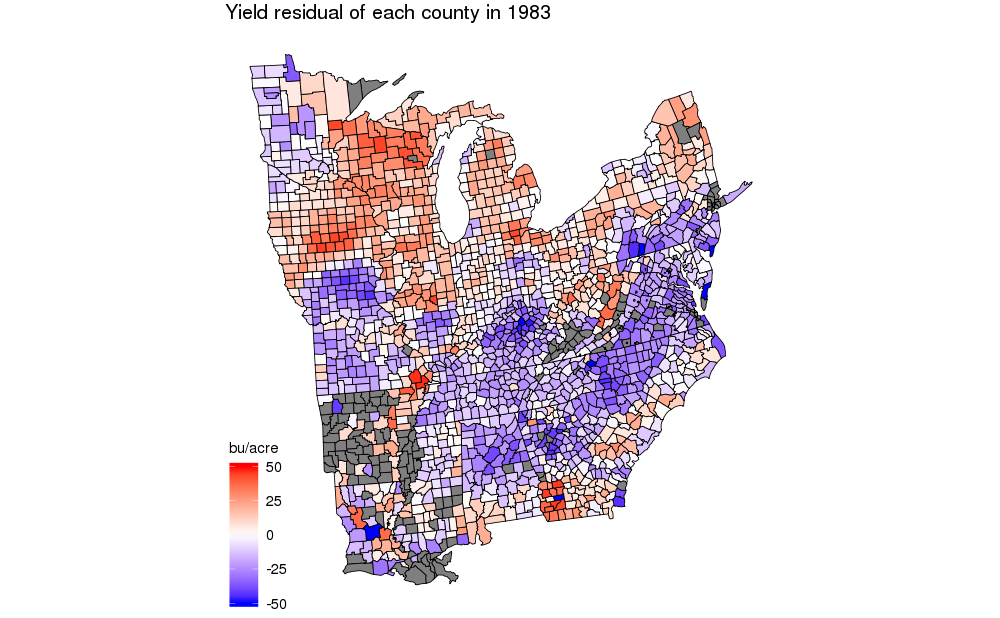

Supplement: S8 Fig — The yield residuals have strong spatial patterns varying each year. We plot the residual map in 1983 with the most observation data. In future studies, we plan to use spatial models to better account for these spatial patterns. (TIF) [file pone.0259180.s008.tif]

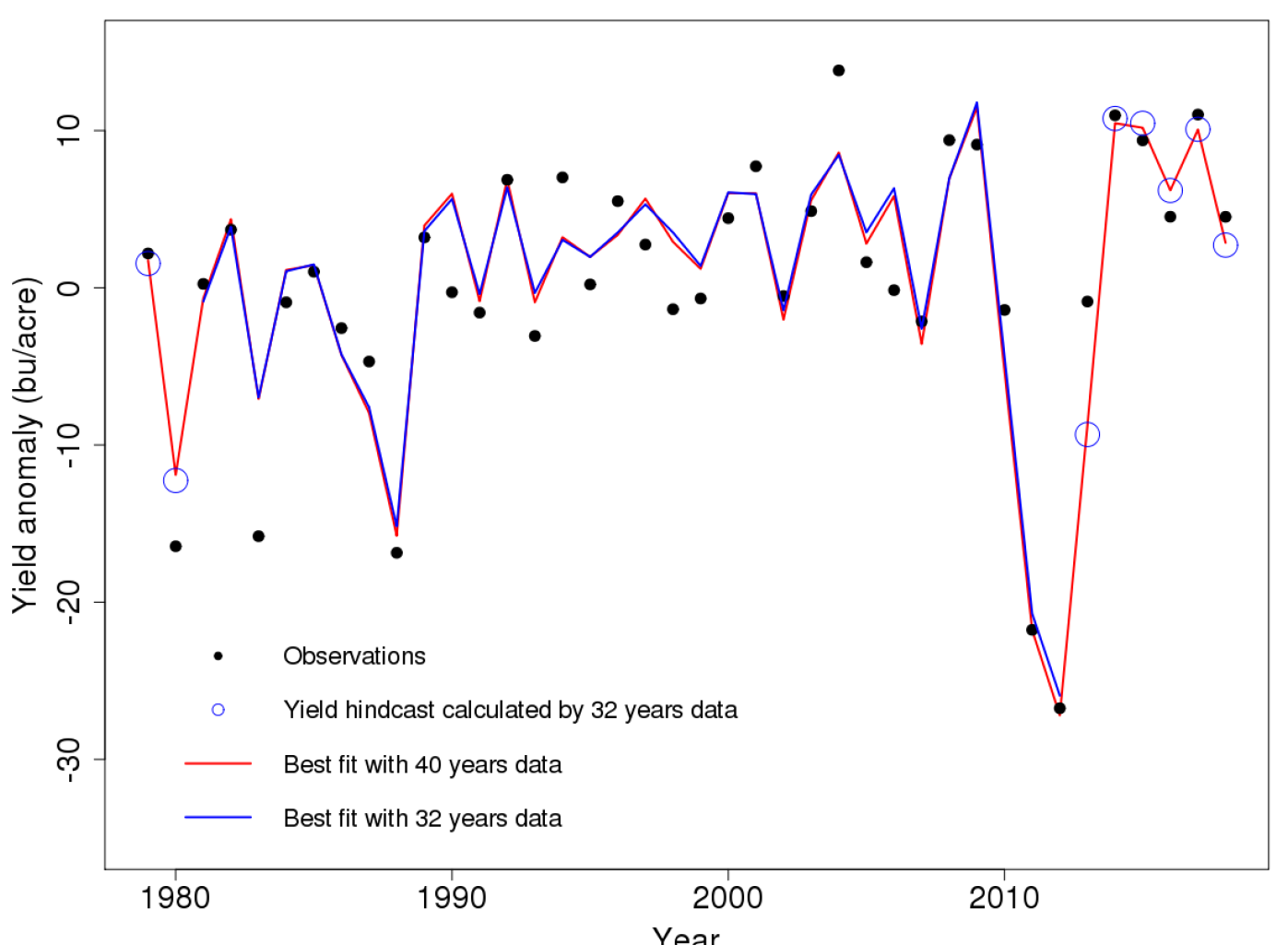

Supplement: S9 Fig — We add eight more years observational data in an update (1979, 1980, 2013–2018). We use these data to test the predictive skill of the old model using 32 years data. The estimated hindcasts given by the old model (blue circles) are close to the hindcasts of the updated model (red line) and the observations (black dots). (TIF) [file pone.0259180.s009.tif]
